# Roux-en-Y gastric bypass contributes to weight loss-independent improvement in hypothalamic inflammation and leptin sensitivity through gut-microglia-neuron-crosstalk

**DOI:** 10.1016/j.molmet.2021.101214

**Published:** 2021-03-16

**Authors:** Jiesi Chen, Nadine Haase, Sven-Bastiaan Haange, Robert Sucher, Julia Münzker, Elisabeth Jäger, Kristin Schischke, Florian Seyfried, Martin von Bergen, Mohammed K. Hankir, Ute Krügel, Wiebke K. Fenske

**Affiliations:** 1 Medical Department III, Endocrinology, Nephrology, and Rheumatology, University Hospital of Leipzig, Leipzig, Germany; 2 Department of Molecular Systems Biology, Helmholtz Center for Environmental Research-UFZ, Permoserstraße 15, 04318 Leipzig, Germany; 3 Division of Bariatric Surgery, Clinic of Visceral, Transplant, Thoracic, and Vascular Surgery, University Hospital, Liebigstraße 20, D-4015, Leipzig, Germany; 4 Department of General, Visceral, Transplant, Vascular, and Pediatric Surgery, University Hospital, Würzburg, Germany; 5 Institute of Biochemistry, Faculty of Life Sciences, University of Leipzig, Talstraße 33, 04103 Leipzig, Germany; 6 Department of Experimental Surgery, University Hospital Würzburg, 97080 Würzburg, Germany; 7 Rudolf Boehm Institute of Pharmacology and Toxicology, University of Leipzig, Härtelstraße 16-18, 04107 Leipzig, Germany; 8 Division of Endocrinology, Diabetes, and Metabolism, Medical Department I, University Hospital of Bonn, Bonn, Germany

**Keywords:** Roux-en-Y gastric Bypass, Bariatric surgery, Hypothalamic inflammation, Endoplasmic reticulum stress, Toll-like receptor 4, Gut microbiota-brain axis

## Abstract

**Objective:**

Hypothalamic inflammation and endoplasmic reticulum (ER) stress are extensively linked to leptin resistance and overnutrition-related diseases. Surgical intervention remains the most efficient long-term weight-loss strategy for morbid obesity, but mechanisms underlying sustained feeding suppression remain largely elusive. This study investigated whether Roux-en-Y gastric bypass (RYGB) interacts with obesity-associated hypothalamic inflammation to restore central leptin signaling as a mechanistic account for post-operative appetite suppression.

**Methods:**

RYGB or sham surgery was performed in high-fat diet-induced obese Wistar rats. Sham-operated rats were fed ad libitum or by weight matching to RYGB via calorie restriction (CR) before hypothalamic leptin signaling, microglia reactivity, and the inflammatory pathways were examined to be under the control of gut microbiota-derived circulating signaling.

**Results:**

RYGB, other than CR-induced adiposity reduction, ameliorates hypothalamic gliosis, inflammatory signaling, and ER stress, which are linked to enhanced hypothalamic leptin signaling and responsiveness. Mechanistically, we demonstrate that RYGB interferes with hypothalamic ER stress and toll-like receptor 4 (TLR4) signaling to restore the anorexigenic action of leptin, which most likely results from modulation of a circulating factor derived from the altered gut microbial environment upon RYGB surgery.

**Conclusions:**

Our data demonstrate that RYGB interferes with hypothalamic TLR4 signaling to restore the anorexigenic action of leptin, which most likely results from modulation of a circulating factor derived from the post-surgical altered gut microbial environment.

## Highlights

• RYGB surgery-related weight loss independently restores hypothalamic leptin signaling and action in diet-induced obesity.
• RGYB modulates hypothalamic TLR4-mediated pro-inflammatory signaling and ER stress to restore leptin's anorexigenic action.
• Humoral factors contribute to modulated microglia-POMC neuron interaction, which appears specific to the RYGB procedure.
• Altering the gut microbiota environment by antibiotics deteriorates leptin's feeding suppressive action after RYGB.

## 1 Introduction

The increasing worldwide prevalence of obesity has become one of the greatest threats to human health. Although strategies based on lifestyle intervention are fundamental for preventing obesity, they provide little benefit as long-term weight-loss solutions [1]. Bariatric surgical procedures, such as Roux-en-Y gastric bypass (RYGB), are recommended as the most effective treatments for morbid obesity and type 2 diabetes [2,3]. However, these procedures are highly invasive, bind clinical capacities, and are limited as a last therapeutic resort to a minor population of patients in need of therapy [4].

While the beneficial outcome of RYGB on adiposity reduction is largely attributed to modulated eating behavior characterized by altered food preference [5] and appetite suppression, the mechanistic basis through which RYGB surgery modulates hunger and satiety sensation remain largely unclear [6].

Preclinical evidence suggests that that RYGB-operated mice exhibit an anorectic hypothalamic neuropeptide profile compared to formerly obese counterparts losing weight due to calorie restriction [7]. This anorectic profile has been reported to be associated with lower hypothalamic activity of the protein tyrosine phosphatase 1B (PTP1B) [8,9], a major negative regulator of leptin receptor signaling in obesity, and with increased activation of leptin receptor downstream signals [8]. Together with further findings obtained from hyperphagic obese melanocortin 4 receptor (MC4R)-deficient [10,11] and leptin-deficient ob/ob mice [12], both of which demonstrate reduced feeding suppression and weight loss compared to wild-type mice receiving RYGB surgery, these data indicate a critical role of the melanocortinergic system for the long-term defense of appetite suppression and lower adiposity following RYGB surgery.

While the system pathway through which RYGB surgery may recruit melanocortinergic signaling is yet unknown, a growing body of evidence suggests that hypothalamic inflammation and cellular stress responses induced by nutrient excess [13] are sensitively linked to compromised neuronal leptin sensitivity [[14], [15], [16], [17], [18], [19]]. Moreover, preclinical studies focusing on the role of the gut microenvironment have shown that gut-derived endocrine factors may reduce microglial activation and hypothalamic inflammatory processes [20] and amplify the catabolic actions of exogenous leptin in obesity [21,22].

In this study, we applied RYGB surgery to a rat model of diet-induced obesity (DIO) to test our hypothesis that appetite suppression following RYGB surgery results from a procedure-specific restoration of hypothalamic leptin receptor sensitivity through modulation of gut microbiota-brain crosstalk.

## 2 Materials and methods

### 2.1 Animals and diets

All the experiments were performed on male age-matched (8- to 10-week-old) Wistar rats (RjHan:WI, Janvier Labs, Le Genest-Saint-Isle, France) that were allocated to the experimental groups based on their body weight. The animals were housed in a specific pathogen-free environment and all of the procedures were conducted according to the international guidelines of animal care and study protocols approved by Landesdirektion Sachsen, Germany, the local governmental authority for animal care (TVV 45/16).

At approximately 8 weeks of age, the animals were placed on a HFD (kcal%: fat, 59%; carbohydrates, 15%; protein, 26%, Diet D12230, Sniff Spezialdiäten GmbH) for eight weeks. The animals were then single-housed and maintained on a strict 12-h light/dark cycle at 22 °C with 50% humidity.

### 2.2 Surgical and post-operative procedures

The surgical protocol was previously described [5]. Briefly, the animals were anesthetized with 3–4% isoflurane in 2 L/min oxygen and maintained on 1.5–2% isoflurane at 0.5 L/min. For RYGB surgery, the jejunum was transected aborally to the pylorus to create a 10 cm biliopancreatic and alimentary limb. The proximal end was anastomosed to the ileum 25 cm orally, creating a common channel for jejuno-jejunostomy. Then the stomach was transected 3 mm below the gastroesophageal junction to create a gastric pouch no larger than 2–3% of the original stomach. End-to-side anastomosis was performed at the proximal end to achieve a gastro-jejunostomy. With the isoflurane concentration reduced to 1.5–2%, abdominal closure was performed. For the sham procedure, gastrointestinal manipulation was conducted via mobilizing the small bowel and gastroesophageal junction and performing a 1-cm-long gastrostomy on the anterior wall of the stomach before the abdomen was closed. Post-operatively the animals were administered carprofen (5 mg/kg, s.c.) for 4 days.

### 2.3 Post-operative diet and monitoring

Directly after surgery, the animals were fed a liquid diet (DietGel Recovery, Clear H_2_O, USA) for 4 days with a subsequent slow adaption to solid chow. Afterward, the sham-operated and RYGB-operated animals were placed on a two-choice diet with standard chow (SC, low-fat diet) and HFD ad libitum, and the body-weight matched group was calorie-restricted to achieve and maintain a similar body weight as the RYGB-operated animals. Following a two-week post-operative recovery, body weight and food intake were monitored twice a week for 12 weeks. For antibiotic cocktail treatment, systemic broad-spectrum antibiotics (ABx) consisting of ampicillin (1 g/L), vancomycin (500 mg/L), neomycin (1 g/L), and metronidazole (1 g/L) according to Thaiss et al. [23] were provided fresh every day through drinking water for 35 days, during which food intake was monitored.

### 2.4 Intracerebroventricular injection

Intracerebroventricular (i.c.v.) injection was performed according to Koch et al. [24]. The sham- and RYGB-operated rats were injected i.c.v. with a single dose of either TAK242 (Millipore, Germany) at 1 μg (in 2 μL of vehicle) or thapsigargin (Sigma–Aldrich, USA) at 10 μg (in 2 μL of vehicle) per day for three days. The same volume of vehicle artificial cerebrospinal fluid (aCSF, Thermo Fisher Scientific, Germany) was injected into control rats. Directly after the last injection, leptin (R&D Systems, USA) was applied i.p. with a dose of 1 mg/kg. Food intake was monitored at 8 h and 24 h after leptin injection. To investigate the role of the RYGB gut microbiota in leptin-induced feeding suppression, the Abx-treated RYGB rats received a single dose of 1 mg/kg of leptin i.p. and their food intake was monitored for 12 h.

### 2.5 Tissue collection

Brain, colon, and ileum tissues used for RNA analysis were carefully removed and immediately frozen in 2-methylbutane cooled on dry ice. For histology, colon and ileum tissues were fixed in 4% paraformaldehyde (PFA) in PBS after removal and subsequently embedded in paraffin. Whole blood was obtained through the portal vein and transferred in Microvette 600 tubes (Sarstedt, Germany). After centrifuging at 9000 *g* for 5 min at 4 °C, plasma samples were collected from the supernatant. Human plasma used in this study was obtained from patients at University Hospital Leipzig. Tissues were stored at −80 °C before further analysis.

### 2.6 Cell culture and treatments

Immortalized BV2 microglial cells were a generous gift from Dr. Angela Schulz of the University Leipzig, Germany. The cells were cultured in RPMI 1640 medium (2 mM of l-glutamine) with 10% heat-inactivated fetal bovine serum (FBS), 100 U/mL of penicillin, 100 μg/mL of streptomycin, and 10 μg/mL of gentamycin (all from Gibco, USA). For BV2 cell experiments with rat plasma, human plasma, and TAK242, the cells were seeded at 1 × 10^6^/well in 6-well plates for protein and mRNA collection, and at 3 × 10^5^/well on glass coverslips in 12-well plates for morphological analysis. The cells were cultured overnight and starved in serum-free medium for 2 h prior to treatments. The supernatant was centrifuged at 1000 rpm for 5 min and filtered to prepare conditioned medium (CM) after 4 h of rat plasma exposure. Adult murine hypothalamic POMC (GFP-1 line) were purchased from CELLutions Biosystems and grown in DMEM (Sigma–Aldrich, Germany) supplemented with 10% fetal bovine serum (FBS), 4.5 g/L glucose and 1% penicillin-streptomycin. The cells were seeded at 1 × 10^6^/well in 6-well plates for mRNA collection and starved in serum-free medium for 2 h prior to treatment. One-third of the medium was replaced with BV2 CM for 4 h or 24 h and RNA was harvested for further analysis. For cell viability tests, POMC cells were seeded at 3 × 10^4^/well in 96-well plates and cultured overnight. After starvation for 4 h, the cells were incubated with BV2 CM medium for 24 h and a CyQUANT XTT Cell Viability Assay (Invitrogen, USA) was performed according to its manual.

### 2.7 Quantitative real-time PCR

The hypothalamus was dissected and ground into power in liquid nitrogen. The total RNA from these tissues was then extracted using an RNeasy mini kit (QIAGEN, Germany) following the manufacturer's instructions. For cell cultures, 1 mL of TRIzol (QIAGEN, Germany) was added to each well for lysis and RNA was isolated according to a standard procedure. One μg of RNA was then applied for reverse transcription using a QuantiTect Reverse Transcription kit (QIAGEN, Germany) according to the manufacturer's protocol. For rat tissues, transcript levels of *Cd68*, *Emr1*, *Gfap*, *Tlr4*, *Myd88*, *Il6*, *Pomc*, *Npy*, *Agrp*, *Cart*, *Bdnf*, *Trh*, *Crh*, *Nlrp3*, *Ptp1b*, *Scos3*, *Mch*, *Tjp1*, and *Ocln* (Supplemental Table 1) were quantified using SYBR Green-based assays (Roche, Germany) on a Roche Light Cycler system. *Gapdh* was used as a reference gene. The purity of the qPCR reaction was controlled through melting curve analysis. Transcription of *Il1b*, *Tnfa*, and *Il18* was evaluated using a TaqMan assay according to its protocol (Thermo Fisher Scientific, Germany). For cell culture experiments, transcript levels of *Iba1*, *Tlr4*, *Md2*, *Myd88*, *Cd14*, *Nlrp3*, *Il18*, *Pomc*, *Obrb*, and *Bdnf* were examined using SYBR Green-based assay as previously mentioned, and *Gapdh* was used as a reference gene (Supplemental Table 2). Transcription of *Il1b*, *Tnfa*, and *Il6* was evaluated using a TaqMan assay according to its protocol. Fold change was determined by ΔΔCt, which was calculated as: 2ˆ - (Ct value gene of interest - Ct value reference gene).

### 2.8 Western blotting assay

Western blotting analysis was performed to examine the expression of GFAP, IBA1, p–NF–κB (p-P65), NF-κB (P65), IκBα, p-eLF2α, eLF2α, p-IRE1, IRE1, p-STAT3, STAT3, p-ERK, ERK, SOCS3, PTP1B, TLR4, Myd88, β-actin, and GAPDH (Supplemental Table 3). Proteins were visualized by an ECL substrate kit (Pierce, USA) and analyzed semi-quantitatively using GeneTools software (Syngene, USA).

### 2.9 Histology

Periodic acid-Schiff (PAS) staining was performed to visualize the number of intestinal goblet cells according to the manufacturer's instructions (Sigma–Aldrich, USA). Colon sections were stained by H&E staining and the villi length was measured with ImageJ (NIH, USA). Immunocytochemistry (ICC) was performed as previously described [19] and analyzed on a fluorescent microscope (Zeiss ApoTome, Germany).

### 2.10 ELISA

Plasma leptin levels were measured by an ELISA kit (Ab100773, Abcam, USA) according to the manufacturer's protocol. Circulating cytokine levels were measured using a V-PLEX Pro-inflammatory Panel 2 Rat kit (MSD, USA). Plasma levels of LPS binding-protein (LBP) were measured using an LBP ELISA kit (HK503, Hycult Biotech, Netherlands).

### 2.11 Analysis of 16S rRNA genes

Frozen cecum content samples were collected and processed for bacterial DNA isolation using a QIAamp DNA Stool Mini kit (Qiagen, Germany) according to the manufacturer's instructions and were then submitted to BGI (Hong Kong, China) (http://www.genomics.cn/en/index) for bacterial 16S DNA V3–V4 amplicon sequencing. Significant differences in alpha diversity indices and the relative abundance of taxa between sample groups were determined by the Kruskal–Wallis test corrected for multi-testing by the Benjamini-Hochberg method [25] followed by post hoc pairwise statistical analysis using the Dunn test. Beta diversity between samples was analyzed by a principal component analysis (PCA) using R, and global significant differences between sample groups were calculated by PERMANOVA using the Adonis function from the vegan R package [26,27]. The figures were constructed using the ggplot2 R package [28].

### 2.12 Statistics

All the statistical analyses were performed using GraphPad Prism software if not otherwise indicated. Data are presented as mean ± SEM unless otherwise stated. Comparisons between two groups were performed using a non-paired two-tailed Student's *t* test. ANOVA was used for comparison among multiple groups followed by pairwise Tukey's post hoc test if not otherwise indicated. Statistical significance was indicated by *P* value.

## 3 Results

### 3.1 Post-RYGB hypophagia was associated with enhanced leptin-induced feeding suppression and hypothalamic leptin signaling in diet-induced obese rats

**Figure 1:**
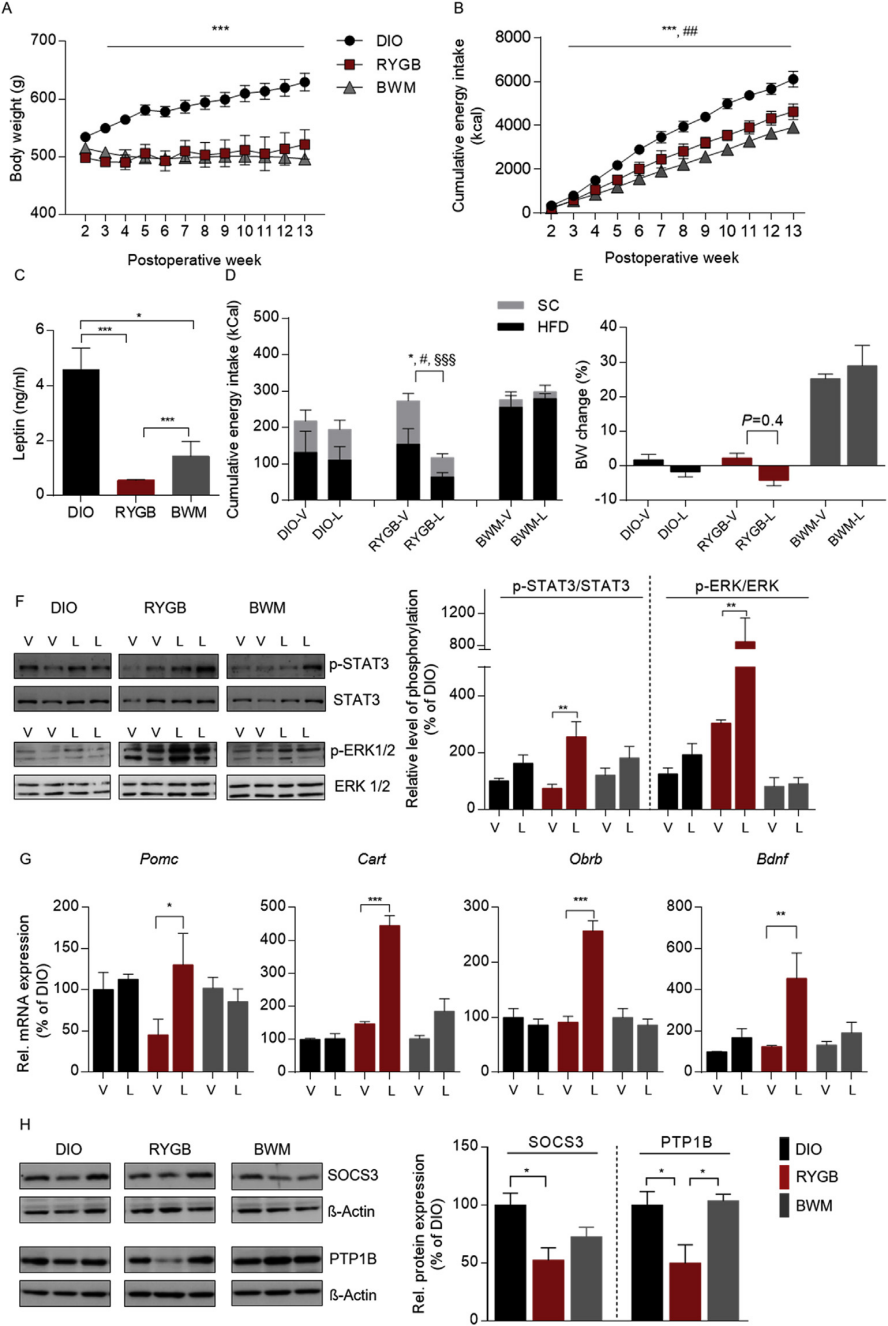
**Post-RYGB hypophagia was associated with enhanced leptin-induced feeding suppression and hypothalamic leptin signaling in diet-induced obese rats.** (A) Weekly post-operative body weights of high-fat (HF) DIO rats that underwent Roux-en-Y gastric bypass (RYGB) surgery compared to sham-operated control groups. Post-operatively, the animals were maintained on a two-choice diet with standard chow (SC) and HFD. (B) Corresponding cumulative energy intake. (C) Plasma leptin concentration (ng/ml) at the twelfth post-operative week. (D–G) To test for leptin sensitivity, during the twelfth post-operative week, the animals were administered either leptin (i.p., 1 mg/kg per day, L) or vehicle (V) for 3 days. (D) Three-day cumulative energy intake of leptin- or vehicle-treated animals. (E) Percent change in body weight during 3-day leptin treatment vs vehicle control. (F) Hypothalamic phosphorylation of STAT3 and ERK at the twelfth post-operative week. (G) Corresponding hypothalamic mRNA expression of *Pomc*, *Cartpt*, *Obrb*, and *Bdbf*. (H) Corresponding protein expression of SOCS3 and PTP1B at 12 weeks post-operatively. Data are presented as mean ± SEM (n = 9–13 for A and B and n = 4–6 for C-G) with individual datapoints. ∗∗∗*P* < 0.001 for the effect of DIO vs RYGB, ##*P* < 0.05 for RYGB vs BWM for A and B; ∗*P* < 0.05 and ∗∗∗*P* < 0.001 for the effect of any indicated comparison for C and H; #*P* < 0.05 for SC intake comparison; ∗*P* < 0.05 for HFD intake comparison; §*P* < 0.05 for cumulative energy intake comparison for D; ∗*P* < 0.05, ∗∗*P* < 0.01, and ∗∗∗*P* < 0.001 for the effect of leptin-vs vehicle-treated animals.

Male DIO rats were allocated to RYGB or sham surgery. To control for the weight loss effect of RYGB, the animals after sham surgery were further randomized to an ad libitum feeding (DIO) or a calorie restriction (CR) group (body weight matched to RYGB, BWM) as previously described [5]. Post-operative body weight and feeding records revealed a similar weight loss maintenance between the RYGB and BWM groups compared to the DIO controls (Figure 1A). Weight loss in both groups was linked to persistent feeding suppression compared to the DIO controls (Figure 1B), although the animals in the BWM group required substantially less energy intake to maintain the same body weight as their RYGB littermates (Figure 1B).

To determine whether spontaneous feeding suppression emerging after RYGB may have derived from improved systemic leptin responsiveness, we found that despite a similar degree of weight loss, circulating leptin levels were substantially lower in the RYGB than in the chronic CR (Figure 1C). To test our hypothesis that RYGB results in improved neuronal responsiveness to circulating leptin, overnight fasted animals received either a bolus of leptin (1 mg/kg, i.p.) or vehicle (20 mM of sterile Tris–HCl, pH 8.0) once daily for 3 days. Notably, RYGB treatment restored the anorexigenic action of exogenous leptin in DIO, whereas chronic CR failed to improve leptin responsiveness (Figure 1D) and maintenance of lower adiposity under free-feeding conditions (Figure 1E).

To determine the post-RYGB enhanced anorexigenic leptin action at the cellular level, we analyzed the leptin-stimulated hypothalamic phosphorylation of signal transducer and activator of transcription (STAT3) and extracellular signal-regulated kinase (ERK) by Western blotting [29]. Upon leptin stimulation, the RYGB rats showed a significant increase in the phosphorylation of STAT3 and ERK (Figure 1F), whereas no comparable effect on STAT3 or ERK phosphorylation was detected in the DIO and CR-treated BWM rats. These data indicated that RYGB treatment facilitated hypothalamic leptin signaling in DIO independent of the reduction in adiposity (Figure 1F).

To identify the central targets of RYGB on appetite control, we studied the influence of RYGB on key hypothalamic signaling networks that orchestrate energy homeostasis. Other than chronic CR, a pronounced upregulation of genes encoding the leptin-regulated anorexigenic peptides proopiomelanocortin (*Pomc*), cocaine-, and amphetamine-regulated transcript prepropeptide (*Cart*), brain-derived neurotrophic factor (*Bdnf*), and the long isoform leptin receptor Ob-Rb (*Obrb*) was found in the RYGB-treated rats in response to leptin administration (Figure 1G). Furthermore, protein and transcript levels of the negative leptin regulators suppressor of cytokine signaling 3 (SOCS3) and protein tyrosine phosphatase 1B (PTP1B), which are upregulated in the hypothalamus following chronic HFD consumption to compromise hypothalamic leptin sensitivity (Milanski et al., 2012; Wu et al., 2014), decreased exclusively after RYGB intervention (Figures. 1H and S1B). Notably, transcripts of the orexigenic neuropeptides remained unaffected by leptin stimulation (Figure. S1A). These data indicated that RYGB intervention mainly affected leptin-directed appetite suppressing pathways.

### 3.2 RYGB surgery reduced hypothalamic gliosis, inflammatory-like responses, and ER stress in DIO rats

**Figure 2:**
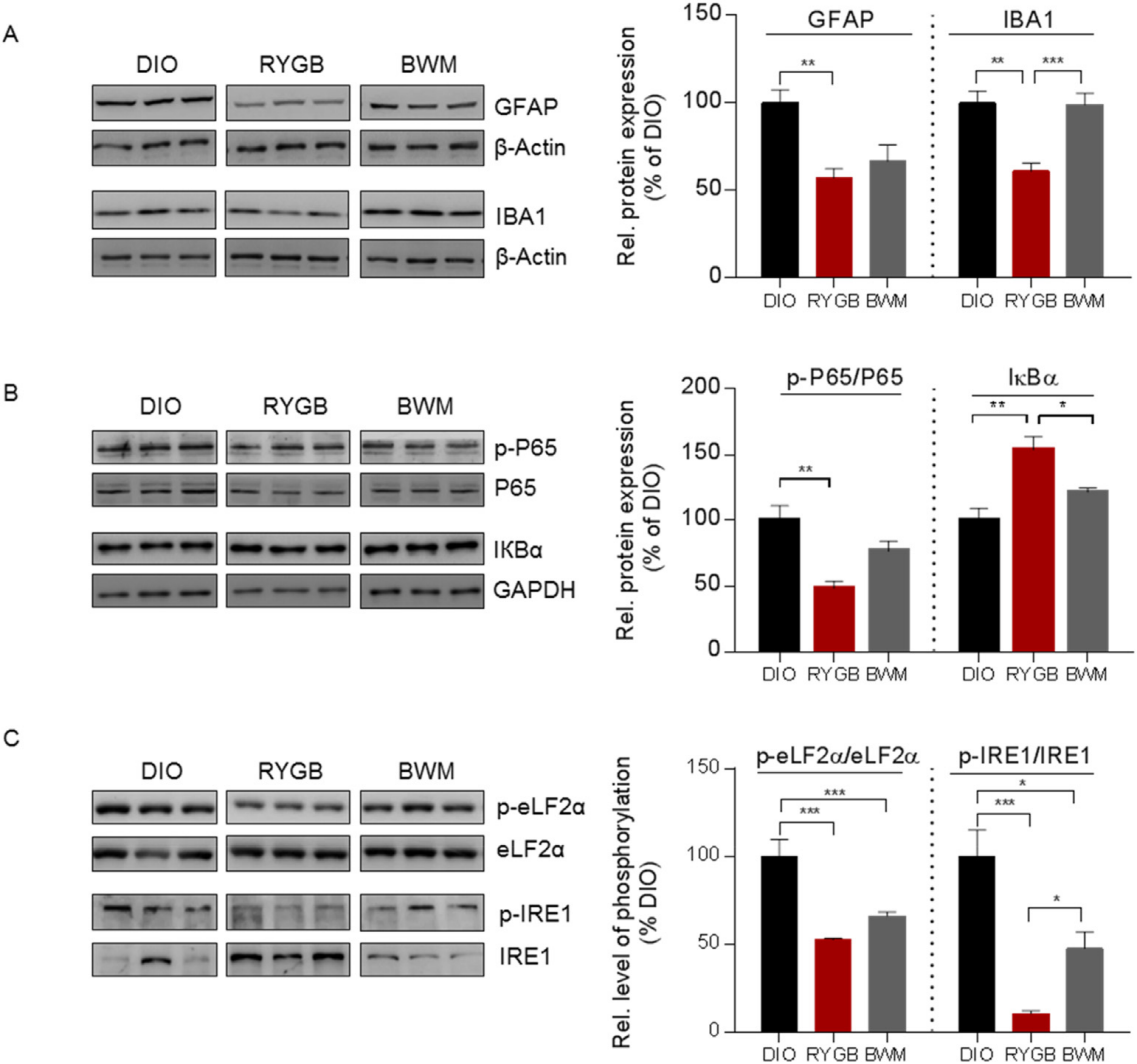
**RYGB surgery reduced hypothalamic gliosis, inflammatory-like response, and ER stress in DIO rats.** (A) Reactive gliosis measured by hypothalamic protein expression of GFAP and IBA1 in RYGB- vs sham-operated DIO and BWM rats at 12 weeks post-operatively. (B) Corresponding phosphorylation of p65 and protein expression of IКBα in the hypothalamus as measured by Western blotting. (C) Corresponding ER stress markers (phosphorylation of eLF2α and IRE1) in the hypothalamus. Data are presented as mean ± SEM (n = 4–6) with individual datapoints. ∗*P* < 0.05, ∗∗*P* < 0.01, and ∗∗∗*P* < 0.001 for the effect of any indicated comparison.

Hypothalamic inflammatory signaling [14] and ER stress [16,30] have been extensively linked to reduced central leptin sensitivity and HFD-induced obesity. But the fundamental question, i.e., whether these are targetable processes qualified to invert the dynamics toward lower energy balance, remains inconclusive. Determining the hypothalamic status of reactive gliosis, NFkB-IKK signaling, and ER stress, we observed a tendency for lower expression of the astrocyte and microglia markers glial fibrillary acidic protein (GFAP) and a strongly reduced expression of ionized calcium-binding adaptor molecule 1(IBA1) (Figure 2A) in the RYGB-treated animals compared to the BWM controls. Consistent with this pattern of reduced glial response, hypothalamic mRNA expression of *Cd68*, *Emr1*, and *Gfap* was exclusively reduced after RYGB- but not CR-induced weight loss (Figure S2A).

IKKβ/NF-KB is a master switch and central regulator of innate immunity [31] that essentially connects overnutrition with compromised leptin signaling in the hypothalamus [14]. We found that the phosphorylation status of the NF-kB p65 unit, an indicator of activated cellular inflammatory response, was downregulated in the hypothalamus specifically after RYGB treatment, whereas this effect was not seen in the BWM counterparts (Figure 2B). Furthermore, the expression of the inhibitor of nuclear factor kappa B (IκBα), which specifically binds to and blocks the nuclear translocation of NF-kB, was upregulated secondary to RYGB treatment, but not CR (Figure 2B). These results were linked to reduced hypothalamic phosphorylation of protein kinases p38 MAPK and SAPK protein specifically following RYGB treatment (Figure S2B).

Another cellular process that critically contributes to hypothalamic leptin resistance [32] and is heavily linked to the IKKβ/NF-kB pathway in diet-induced obesity is ER stress [14,33]. By determining whether RYGB intervention interferes with the ER stress response to improve hypothalamic leptin signaling, we found that hypothalamic ER stress, as assessed by the phosphorylation levels of eukaryotic initiation factor 2 α-subunit (eLF2α) and inositol-requiring enzyme 1α (IRE1), was reduced in both weight-loss groups compared with the DIO controls (Figure 2C). These data indicated a state of less activated unfolded protein response (UPR) secondary to weight loss. Of note, although chronic CR also reduced phosphorylation levels of both proteins, this effect was clearly less pronounced than after RYGB treatment. Consistent with that, mRNA expression of UPR markers binding immunoglobulin protein (*Bip*), C/EBP homologous protein (*Chop*), and activating transcription factor 4 (*Atf4*) were exclusively reduced secondary to weight-loss surgery (Figure. S2C). Together, these data demonstrated that RYGB surgery exerts robust and procedure-specific effects in relieving hypothalamic inflammatory and ER stress in HFD-induced obesity.

### 3.3 RYGB surgery modulated hypothalamic TLR4 signaling and ER stress to restore leptin's anorexigenic action

A critical mechanism by which the nutritional environment directly influences immune cell signaling and ER stress in the central nervous system is through recognition by pathogen-sensing molecules [18,19]. This includes signaling through toll-like receptors (TLRs), among which activation of TLR4 expressed in innate immune cells plays a key role in HFD-induced hypothalamic inflammation and metabolic disease [[34], [35], [36]]. Rodents with pharmacological inhibition and knockdown of TLR4 as well as neuronal deletion of the TLR adaptor molecule myeloid differentiation primary response 88 (MyD88) are protected from HFD-induced leptin resistance and metabolic disorders [34,35,37,38]. However, the role of TLR4 in RYGB-mediated recovery of central leptin sensitivity and feeding suppression is unknown.

**Figure 3:**
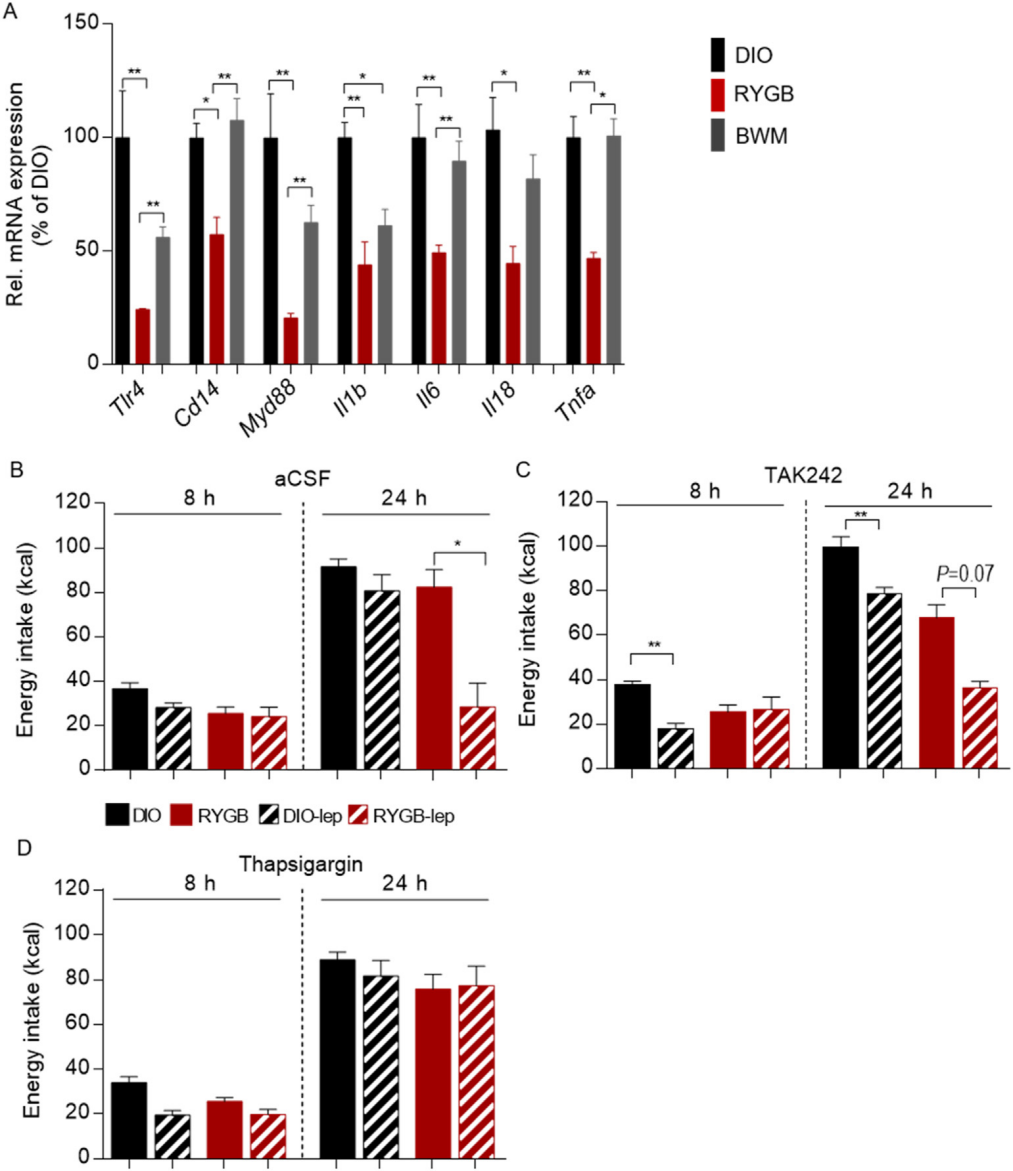
**RYGB surgery modulated hypothalamic TLR4 signaling and ER stress to restore leptin's anorexigenic action.** (A) Gene expression of *Tlr4*, *Myd88*, *Cd14*, *Il1b*, *Il6*, *Il18*, and *Tnfa* in the hypothalamus of DIO rats that underwent RYGB surgery compared to sham-operated control groups at 12 weeks post-operatively. (B–D) RYGB- and sham-operated DIO animals at the twelfth post-operative week were intracerebroventricularly (i.c.v.) injected with TLR4 inhibitor TAK242 (1 μg per day), ER stress inducer thapsigargin (10 μg per day), or artificial cerebrospinal fluid (aCSF, 2 μL) for three days. On the third day, TAK242, thapsigargin, or vehicle was administered 8 h before administration of leptin (1 mg/kg, i.p.) or vehicle control. Energy intake (kcal) during 8-h and 24-h periods following leptin/control injections. Data are presented as mean ± SEM (n = 3–6, as indicated) with individual datapoints. ∗*P* < 0.05 and ∗∗*P* < 0.01 for the effect of any indicated comparison for A and effect of leptin-vs vehicle-treated animals for B–D.

In this study, we found that hypothalamic transcript levels of *Tlr4*, *Myd88*, and *Cd14* as well as downstream pro-inflammatory cytokines including *Il1b*, *Il6*, *Il18*, and *Tnfa* were exclusively reduced in the RYGB-treated animals (Figure 3A), suggesting a RYGB-specific attenuation of TLR4 signaling. To test in vivo whether reduced hypothalamic TLR4 signaling serves as a mechanistic account for improved leptin responsiveness after RYGB surgery, we administered TAK242 (1 μg/d), a specific TLR4 inhibitor [39], or aCSF (2 μL/d) centrally (i.c.v.) to the RYGB- vs sham-treated rats for three days. Following this period, exogenous leptin administration (10 mg/kg, i.p.) led to significantly reduced energy intake over 8 and 24 h in the DIO rats pretreated with TAK242 (48.0% at 8 h and 78.9% at 24 h vs vehicle, Figure 3B and C). Of note, the same dose of leptin failed to induce additional feeding suppression secondary to TAK242 pre-treatment in the RYGB-treated group (Figure 3B and C). Furthermore, the anorexigenic action of leptin in the RYGB-treated group was offset by pre-treatment with the ER stress inducer thapsigargin (10 μg/d i.c.v. for three days) (Figure 3D), whereas the same pre-treatment showed no effect on feeding in the DIO controls (Figure 3B and D). The findings that central inhibition of TLR4 signaling failed to complement the beneficial effects of surgery on leptin's anorexigenic action, whereas central ER stress induction completely abrogated the restoring effect of RYGB treatment on leptin responsiveness, indicating that RYGB intervention essentially engaged reduced hypothalamic TLR4 signaling and ER stress for its improvement in neuronal leptin receptor signaling and feeding suppression.

### 3.4 RYGB surgery modulated microglia and POMC neuron interaction by a humoral factor

**Figure 4:**
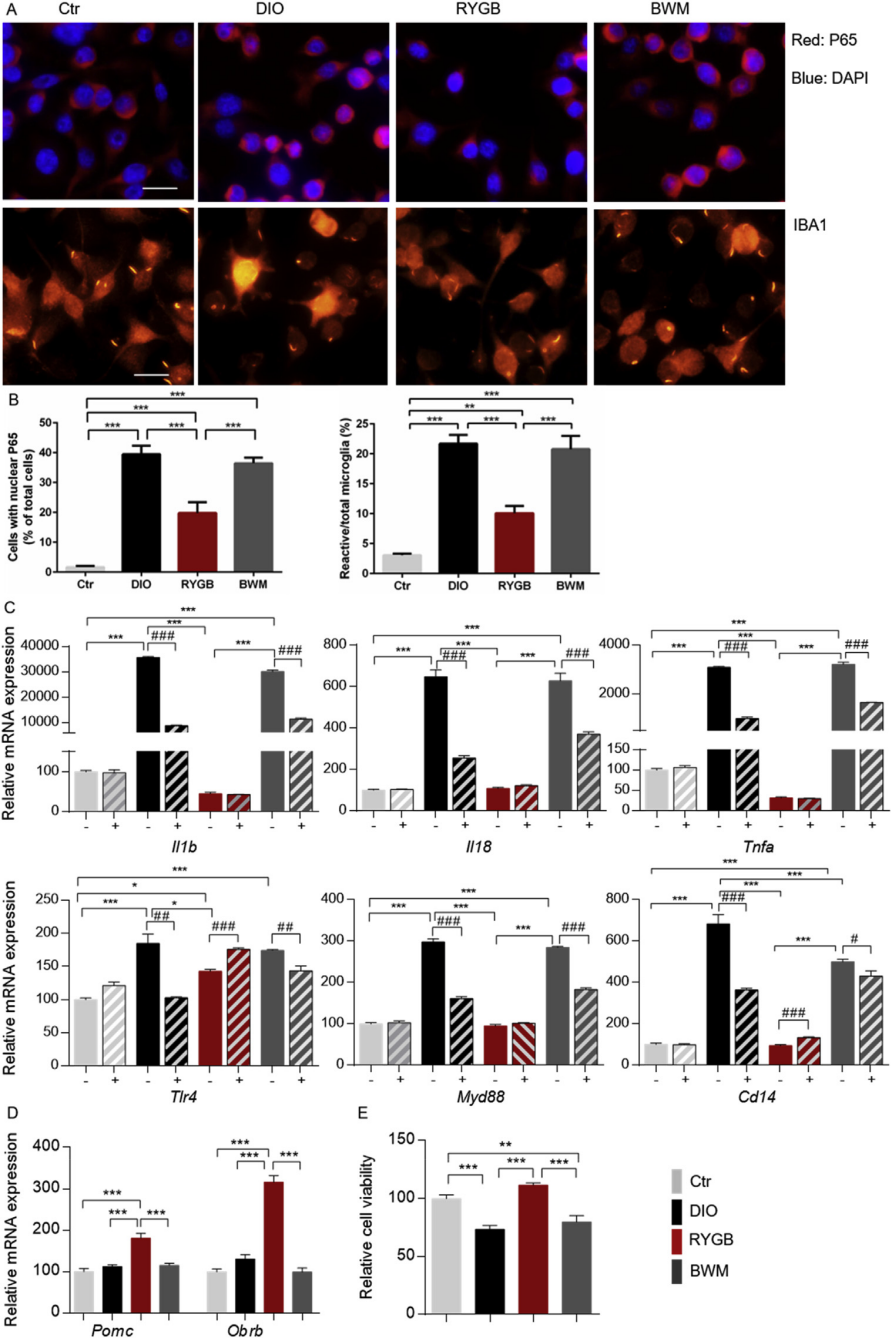
**RYGB surgery modulated microglia and POMC neuron interaction by a humoral factor.** (A and B) BV2 cells were 4-h treated with plasma (2%) collected from RYGB- and sham-operated DIO and BWM rats at 12 weeks post-operatively. (A and B) NF-kB nuclear translocation (upper panel) in cells was assessed by immunofluorescent staining with an anti–NF–kB antibody (red) and DAPI (blue), while morphology was visualized by IBA1 fluorescent staining (lower panel), scale bar, 50 μm (A) and quantification by cell counting of immunofluorescent staining (B). (C) BV2 cells were pretreated by 100 nM TAK242 (+) or vehicle (−) for 1 h before further incubation with medium containing 2% rat plasma for 4 h. The mRNA expression of *Il-1b*, *Il18*, *Tnfa Tlr4*, *Myd88*, and *Cd14* was analyzed by qPCR. (D) Murine hypoPOMC neurons were incubated for 4 h with conditioned medium (CM) obtained from BV2 cell culture that was exposed to rat plasma (2%, 4 h). The mRNA expression of *Pomc* and *Obrb* was assessed by qPCR. (E) POMC neurons were incubated with the same CM for 24 h and cell viability was measured by a CyQUANT XTT Cell Viability Assay. Data are presented as mean ± SEM (n = 3–4) with individual datapoints. ∗*P* < 0.05, ∗∗*P* < 0.01, and ∗*P* < 0.001 for the effect among non-TAK242 pretreated cultures; ###*P* < 0.001 for the effect of TAK242 vs vehicle pretreated cultures for C; ∗∗*P* < 0.01 and ∗∗∗*P* < 0.001 for the effect of any indicated comparison for D and E.

To determine whether the effects of RYGB surgery on microglial activity and central innate immune response resulted from modulated circulating signaling, we treated BV2 microglial cells with plasma collected from the three study groups and visualized NF-kB/65 nuclear translocation in these cells by immunofluorescent staining. In contrast to the control culture, where NF-κB p65 (red) was distributed in the cytoplasm (Figure 4A, upper lane) and cells displayed a typical ramified morphology (Figure 4A, lower lane), cells treated with plasma from sham-operated DIO rats showed increased p65 and DAPI (blue) co-localization, which indicates p65 nuclear translocation, a sign of activated inflammatory response (Figure 4A,B). Consistently, the number of amoeboid-shaped activated microglia increased accordingly (Figure 4A, lower lane, and 4B). Similar morphological changes as seen with DIO were observed in microglia cells treated with plasma from the BWM controls. Of note, treatment of cells with RYGB plasma effectively prevented p65 nuclear translocation, while the number of activated microglia and morphology resembled that of the control group (Figure 4A,B). In line with that, the mRNA expression of the downstream pro-inflammatory cytokines *Il1b*, *Il18*, and *Tnfa* was considerably increased in microglial cells treated with plasma from the DIO and CR-treated animals, but remained at control levels after incubation with plasma obtained from the RYGB-treated animals (Figure 4C).

To further explore whether the observed immune cell response was mediated via microglial TLR4 signaling, BV2 cells were pretreated 1 h prior to plasma exposure with TAK242 (100 nM). While the mRNA expression of *Tlr4*, *Myd88*, *Cd14* and downstream pro-inflammatory cytokines was upregulated after incubation with plasma from the DIO and BWM rats, TAK242 pre-treatment largely prevented this response (Figure 4C). Notably, TAK242 pre-treatment had no effect on pro-inflammatory gene expression in cultures incubated with plasma from the RYGB-operated rats (Figure 4C). Together, these data indicated that a DIO-derived circulating factor may stimulate TLR4-mediated microglial activity and cytokine production and that RYGB intervention may eradicate this signaling molecule from the circulation in a weight-loss-independent manner.

As hypothalamic POMC neurons present the major target for leptin's feeding suppressing effects (Figure 1E), we next determined how humoral modulation of microglial activity influences POMC neuronal activity. After incubation of hypothalamic POMC neurons derived from adult POMC-eGFP transgenic mice (mHypoA-POMC/GFP1 cell line) neurons [40] for 4 h with conditioned medium (CM) harvested from plasma-treated BV2 cells, intriguingly, an upregulation of *Pomc* and *Obrb* mRNA expression was found exclusively in neurons incubated with CM obtained from RYGB plasma pretreated cultures (Figure 4D). Prolonging the incubation time to 24 h, reduced viability of POMC cells was found in cultures treated with CM from DIO and BWM plasma-stimulated BV2 cells, while POMC cells treated with RYGB plasma-stimulated CM were prevented from this effect (Figure 4E). Together, these data indicated that output functions of appetite-curbing POMC neurons are directly linked to microglial activity, which underlies different systemic control following RYGB- vs CR-mediated weight loss intervention.

### 3.5 Reduced LPS-binding protein and systemic pro-inflammatory tone were associated with a distinct shift in gut microbiota from RYGB surgery

Based on our preliminary results pointing toward a scenario where post-RYGB depletion of TLR4 activating signal(s) in the circulation may promote reduced hypothalamic microglial activity and pro-inflammatory tone, we next aimed to specify the circulating signaling candidate.

Although disturbed bacterial signaling to the nervous system through toxins, cytokines, and metabolites provides a hallmark of obesity development [41], the reversibility of this process is still a matter of debate. The bacterial lipopolysaccharide (LPS), an endotoxin present in the outer cell membrane of gram-negative bacteria, is a potent and endogenous TLR4 agonist that binds to the TLR4-MD2 complex and initiates downstream inflammatory responses via bacterial translocation through the intestinal tract.

**Figure 5:**
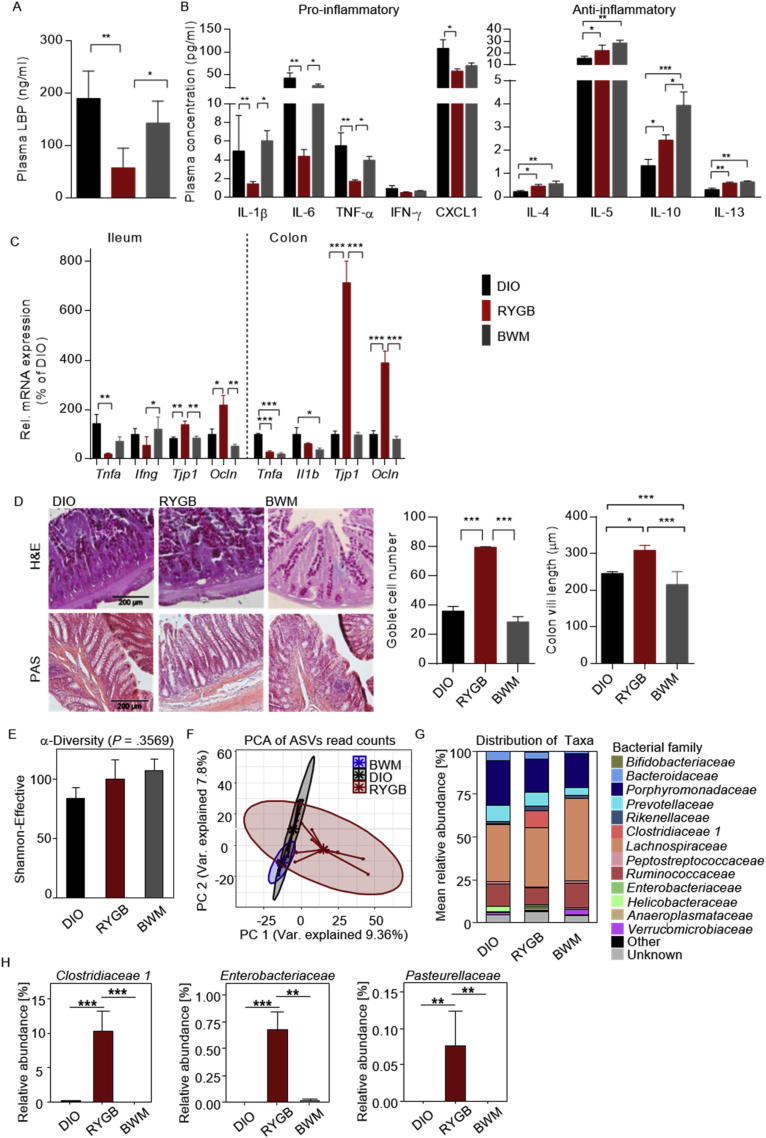
**Reduced LPS-binding protein and systemic pro-inflammatory tone was associated with a distinct shift in gut microbiota from RYGB surgery.** (A) Plasma LPS-binding protein (LBP, ng/ml) levels at 12 weeks post-operatively. (B) Corresponding plasma pro- (IL-1β, IL6, TNF-α, INF-γ, and CXCL1) and anti-inflammatory (IL-4, IL-5, IL-10, and IL13) cytokine levels. (C) Relative mRNA expression of pro-inflammatory cytokines (*Tnfa*, *Ifng*, and *Il1b*) and markers of epithelial barrier integrity (*Tjp1* and *Ocln*) in the ileum and colon of RYGB- and sham-operated DIO and BWM rats at 12 weeks post-operatively. (D) H&E and PAS staining of colon sections (scale bars, 200 μm) and morphometric quantification of villi length and goblet cell count per crypt. (E) Alpha diversity analysis profiles based on a principal component analysis (PCA) of 16S rRNA gene profiling data from cecal content collected from animals at 12 weeks post-operatively. (F) Beta diversity of microbiome (pairwise PERMANOVA, DIO vs RYGB = 0.001, DIO vs BWM = 0.011, and RYGB vs BWM = 0.001). (G) Mean abundance of bacterial families. (H) Significantly altered taxa between study groups. Data are presented as mean ± SEM (n = 3–6 for A-D, n = 6–9 for E–H) with individual datapoints, ∗*P* < 0.05, ∗∗*P* < 0.01, and ∗∗∗*P* < 0.001 for the effect of any indicated comparison.

In this study, we found reduced levels of plasma LPS-binding protein (LBP), an acute phase protein that reflects systemic bacterial endotoxin levels [42,43] following RYGB treatment (Figure 5A), which is associated with decreased concentrations of circulating pro-inflammatory cytokines (Figure 5B). Similar changes in the peripheral inflammatory tone were recently reported in patients after RYGB surgery that were associated with reduced radiologic measures of hypothalamic inflammation [44]. Importantly, these changes in systemic low-grade inflammation and endotoxin levels were specific to RYGB-induced weight reduction, but were absent under chronic CR (Figure 5A and B). In line with that, downregulated expression of intestinal inflammatory markers and increased expression of tight junction markers were found in the ileum and colon of the RYGB-treated rats (Figure 5C). These findings were accompanied by a higher count of mucin-producing goblet cells and an increase in colon villi length after RYGB intervention (Figure 5D), indicating strengthening of intestinal barrier function following this surgery.

As the gut microbiota plays a pivotal role in intestinal barrier integrity [[45], [46], [47], [48]], we wondered whether conserved shifts in the gut microbiota from RYGB surgery [[49], [50], [51]] may promote the remission of metabolic endotoxemia and systemic pro-inflammatory signaling after the procedure. Profiling cecal microbiota communities by 16S rRNA gene amplicon sequencing revealed no difference in alpha diversity among the treatment groups (Figure 5E), whereas a principal component analysis (PCA) of 16S rRNA gene sequencing reads showed substantial differences between microbiota content as a function of treatment with the most diverse microbiome profile found in the RYGB-treated group (Figure 5F).

Changes in the distribution of bacterial families and genera further confirmed a pronounced effect of RYGB intervention on the obese gut microbiome (Figuress. 5G and S3A) with significant differences in taxonomic relative abundance at the family level found for *Clostridaceaea_1*, *Enterobacteriaceae*, and *Pasteurellaceae* (Figure 5H). Similar findings were previously reported post-RYGB in patients and rodents (Paganelli et al., 2019; Shao et al., 2017a). Clostridiales are known as important players for maintaining gut homeostasis [52], which have been associated with improved intestinal dysbiosis and reduced inflammation [[53], [54], [55]]. Interestingly and in line with our findings, the *Clostridaceaea_1* family in this bacterial order has been reported to promote intestinal mucus thickening [56]. Thus, an enriched abundance in this family after RYGB intervention is in accordance with improved intestinal barrier function.

### 3.6 RYGB surgery regulated hypothalamic feeding suppression and leptin's anorexigenic action in a gut microbiota-dependent manner

**Figure 6:**
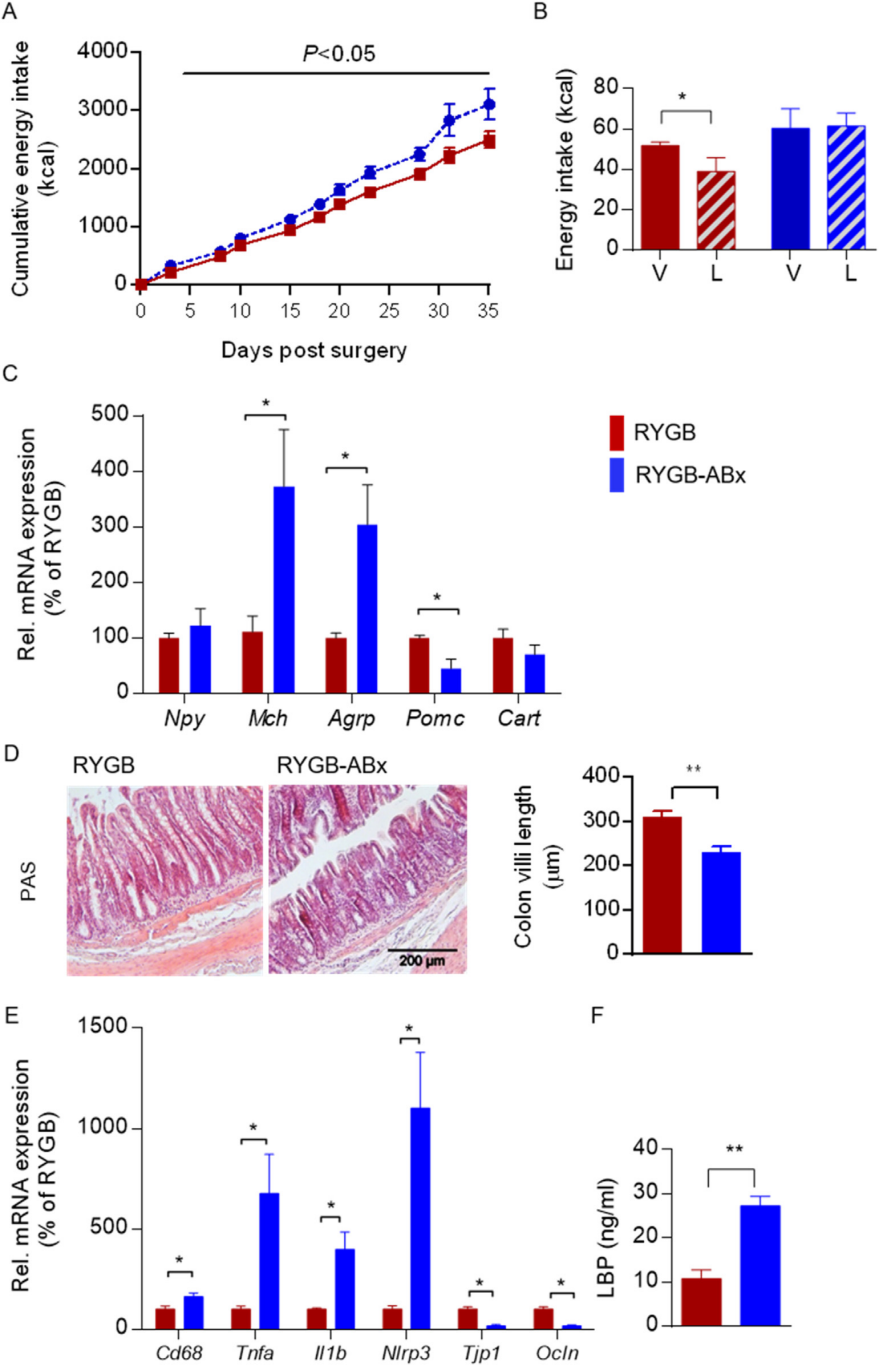
**RYGB surgery regulated hypothalamic feeding suppression and leptin's anorexigenic action in a gut microbiota-dependent manner.** (A–F) RYGB-operated DIO rats at 5 weeks post-operatively were administered antibiotics (ABx) via drinking water for 5 weeks (RYGB-ABx). (A) Cumulative energy intake (kcal) after ABx (RYGB-ABx) vs control (RYGB) over a 35-day period (n = 9–11). (B) At 4 weeks after starting ABx, the animals received a single dose of leptin (i.p., 1 mg/kg, L) or vehicle (V) before 24-h energy intake (kcal) was recorded (n = 6). (C) Hypothalamic mRNA expression of *Npy*, *Mch*, *Agrp*, *Pomc*, and *Cart* (n = 4–6). (D) H&E staining of colonic sections (scale bar, 200 μm) and morphometric quantification of villi lengths (n = 3). (E) Relative mRNA expression of pro-inflammatory (*Cd68*, *Tnfa*, *Il1b*, and *Nlrp3*) and epithelial barrier integrity markers (*Tjp1* and *occludin*) in the colon of RYGB- vs RYGB-ABx-treated DIO rats (n = 3–6). (F) Plasma LPS-BP (ng/ml) levels (n = 3–6). Data are presented as mean ± SEM (n = 3–11 as indicated) with individual datapoints. ∗*P* < 0.05, ∗∗*P* < 0.01, and ∗∗∗*P* < 0.001 for the effect of leptin-vs vehicle-injected animals (B) and RYGB- vs RYGB-ABx-treated DIO rats (C–E).

To functionally study the role of RYGB-altered gut microbiota in the procedure-specific reduction in circulating endotoxins and restoration of leptin-induced feeding suppression, we treated RYGB rats at a stage of stabilized weight reduction with an antibiotic cocktail (ABx) for 5 weeks. Notably, the 5-week microbiota depletion cancelled the beneficial effects of surgery on feeding suppression (Figure 6A) and restored sensitivity to leptin's anorexigenic action (Figure 6B). This outcome was in accordance with a dominant orexigenic gene profile in the hypothalamus (Figure 6C) as well as a relapse in intestinal integrity (Figure 6D and E) and metabolic endotoxemia (Figure 6F) in the RYGB-operated animals under ABx (RYGB-ABx). These findings demonstrated that the gut microbiota contributes to restored leptin sensitivity and feeding suppression resulting from RYGB surgery, in which bacteria-derived endotoxins may constitute a functional player.

### 3.7 Human plasma modulated pro-inflammatory response in microglia in a procedure-specific and weight-loss-independent manner

**Figure 7:**
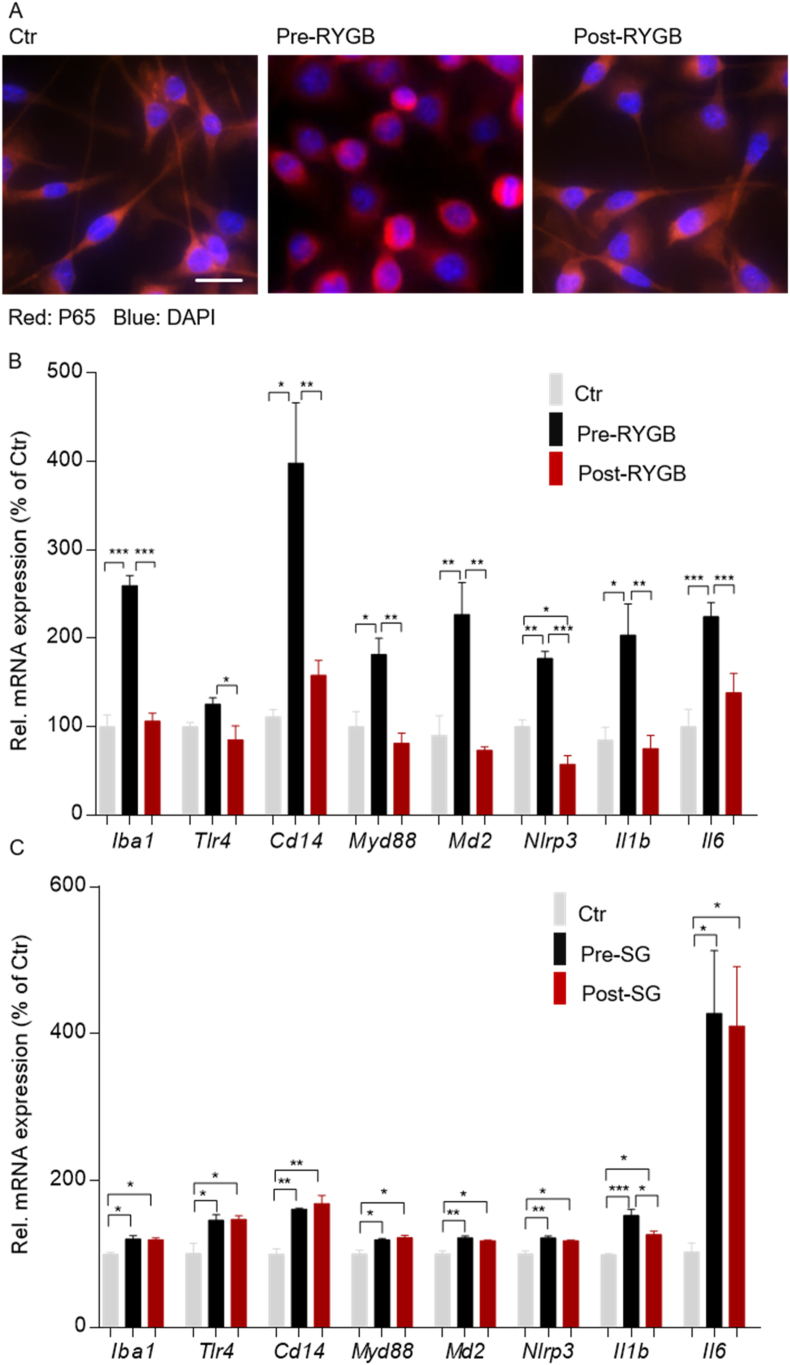
**Human plasma modulated pro-inflammatory response in microglia in a procedure-specific and weight-loss-independent manner.** (A) BV2 cells treated with plasma (2%) from two obese patients before (pre-RYGB, BMI 55.4 and 34.8) and 24 months after RYGB surgery (post-RYGB, BMI 29.2 and 25.1) for 4 h were analyzed for p65 nuclear translocation by immunofluorescent staining (scale bar, 50 μm). (B) The mRNA expression of *Iba1*, *Tlr4*, *Cd14*, *Myd88*, *Nlrp3*, *Il1b*, and *Il6* was analyzed by qPCR. (C) BV2 cells treated with plasma (2%) collected from two obese patients before (pre-SG, BMI 51.9 and 63.7) and 24 months after sleeve gastrectomy surgery (post-SG, BMI 31.1 and 37.5) for 4 h were analyzed for mRNA expression of *Iba1*, *Tlr4*, *Cd14*, *Myd88*, *Nlrp3*, *Il1b*, and *Il6* detected by qPCR. Data are presented as mean ± SEM (n = 3–4) with individual datapoints. ∗*P* < 0.05, ∗∗*P* < 0.01, and ∗∗∗*P* < 0.001 for the effect of any indicated comparison.

To determine the transferability of our findings from rodents to human obesity, we incubated immortalized microglia cells with plasma received from obese patients before (BMI 55.4 and 34.6 kg/m^2^) and 24 months after RYGB treatment (BMI 29.2 and 25.1 kg/m^2^). Cells treated with plasma from obese patients before surgery developed a clearly detectable NF-kB/p65 nuclear translocation (Figure 7A), whereas this effect was absent in cells incubated with plasma from the same patients obtained 24 months post-RYGB surgery (Figure 7A). In line with that, BV2 cells treated with plasma from patients before surgery promoted a significant increase in *Iba1*, *Tlr4*, *Cd14*, *Md2*, *Myd88*, *Nlrp3*, *Il1b*, and *Il6* mRNA expression, whereas BV cells were protected from this effect when treated with plasma obtained from the same patients 24 months after RYGB surgery (Figure 7B). To control for procedure-specificity, we next treated BV2 cells with plasma received from obese patients before (BMI 51.9 and 63.7 kg/m^2^) and 24 months after having undergone sleeve gastrectomy (SG) (BMI 31.3 and 37.5 kg/m^2^). Interestingly, we found that treatment of cells with plasma obtained from patients having experienced the same percentage of weight loss by SG failed to prevent microglial activity and activation of TLR4 signaling (Figure 7C). In line with our preclinical data, these findings demonstrated that human obesity is associated with a humoral factor that contributes to microglial activity and pro-inflammatory response and that RYGB surgery eradicates this humoral effect in a weight-loss-independent but intervention-specific manner.

## 4 Discussion

In this study, we provided new evidence of a casual role of altered gut-brain communication in improved appetite control following RYGB surgery. More specifically, we demonstrated that RYGB treatment of HFD-induced obese rats attenuated microgliosis and inflammatory-like response in the hypothalamus and restored neuronal sensing to the anorexic action of leptin in a TLR4-dependent manner. Additionally, our data indicated that reduced systemic pro-inflammatory signaling derived from the post-RYGB gut microenvironment was mechanistically linked to the suppression of hypothalamic TLR4 signaling and restored leptin responsiveness, and that this effect dissociated from weight loss and reduced HFD intake.

Weight-loss surgical procedures such as RYGB appear unique in their capacity to modulate central immune cell homeostasis despite ongoing access to a HFD. While direct and indirect evidence from rodent [57] and human studies [44,58] supports this concept, the mechanistic basis of how surgical gut rearrangements may control the central innate immune system interacting with neuronal appetite control remains unclear.

Our present data point to hypothalamic TLR4/MyD88 activity as a critical molecular candidate in this scenario. As a key player in the innate immune system and upstream mediator of IKKβ/NF-kB signaling and ER stress [34], previous data reported an essential role of HFD-induced TLR4/MyD88 activity in hypothalamic microglia as an initiating signaling event in leptin resistance and DIO [18,34,37]. Furthermore, brain-specific MyD88 deletion resulting in IKKβ/NF-kB silencing has been reported to protect mice against the development of DIO and restore leptin sensitivity [37] as was demonstrated for pharmacological inhibition of ER stress [14,16,[59], [60], [61]].

As a critical extension to previous data, we report that the central administration of the pharmacological TLR4 inhibitor TAK242 can restore impaired leptin responsiveness in HFD rats, while the same intervention failed to induce additive effects on leptin-induced feeding suppression upon RYGB treatment. Moreover, we demonstrated that while central administration of the ER stress inducer thapsigargin fully abolished the restoring effect of RYGB on leptin-induced feeding suppression, same treatment had no effect on leptin-unresponsive HFD rats. Together, this acute switch in leptin responsiveness upon pharmacological modulation of central TLR4 signaling and ER stress emphasizes that both signaling pathways are fully engaged and essential for improved leptin-induced feeding suppression following RYGB surgery.

Although justified concerns have been raised on whether impaired or rather increased cellular leptin signaling may promote the inability of leptin to suppress feeding in the face of obesity [62,63], there is convincing evidence that amplifying leptin responsiveness using different molecular compounds [17,30,64] markedly reverses hyperphagia and adiposity in experimental obesity. These approaches regenerate the hope of developing leptin's potential as an effective anti-obesity compound.

Notably, we demonstrated that ameliorated hypothalamic inflammatory signaling in RYGB is directly regulated by circulating factors presumably derived from the altered gut microenvironment specific to RYGB intervention. These results may provide a novel direct link between the altered gut microbial composition, the tone of the central immune response, and improved appetite control following RYGB treatment. More specifically and supporting an earlier study relating gut microbiota with central leptin signaling [65], our data herein demonstrate that functional shifts in the gut microbiota resulting from RYGB gut reconfiguration [51,66] may retrieve hypothalamic leptin sensitivity by relieving humoral activation of microglial TLR4 pro-inflammatory signaling in the hypothalamus.

Previous observational studies in rodent [[67], [68], [69]] and human obesity [70] revealed a post-RYGB reduction in metabolic endotoxemia and LPS receptor-mediated inflammatory stress, which was further reported to be associated with post-RYGB with compositional microbiota alterations in obese patients with T2D [71]. Of note, similar findings with respect to the modulated gut microbiota composition after RYGB surgery were recently reported [69]. Interestingly however, the authors found no comparable taxonomic alterations in sham- and SG-operated control animals. This finding is of interest as it is in line with the absent reduction in circulating endotoxin levels reported after SG surgery [72] and fits our present data, which point to a procedure-specific modulation of microbial translocation as a rationale for reduced TLR4-mediated microglial activity and restored neuronal leptin sensitivity promoting appetite suppression following RYGB surgery.

While we demonstrated a RYGB-specific reduction in circulating LBP levels that is linked to reduced TLR4-mediated pro-inflammatory tones, additional data are needed to formally conclude this link. Interestingly, a lipoprotein-mediated transport mechanism in blood–brain interfaces was recently reported [73]. Furthermore, circumventricular organs such as the arcuate nucleus as a major site of leptin action are particularly vulnerable to the pro-inflammatory response mediated by bacterial LPS [74,75]. In support of these findings, prolonged systemic exposure to LPS in normal weight rats was recently demonstrated to induce hypothalamic metainflammation and central insulin resistance [76]. Although findings from systemically LPS-treated rats that developed hyperphagia and showed inhibited leptin signaling in vagal afferent neurons [77] may support our concept, additional mechanisms may modulate hypothalamic immune signaling and remain to be studied.

Moreover, our present data indicated a potentially different mechanism of action on appetite suppression for SG than for RYGB. Interestingly, this outcome was in line with previous data, which found no effect of SG on improved leptin sensitivity [78]. These findings may have important implications and demand further investigation of the fundamental mechanistic differences between both surgical procedures. More detailed functional analyses of gut microbes and their derivates that regulate innate immune responses, neurotransmission, and blood–brain barrier function in a procedure-specific manner may provide valuable insights into the different mechanisms of surgery-induced appetite control. Herein, chemostat culture systems provided a valuable method to in vitro characterize and modulate the gut microenvironment [79], which interacts with the host innate immune system to control neuroinflammation. More importantly, as CNS-targeted therapies are difficult to accomplish, identifying integrated microbiome-based therapeutics recruiting downstream signaling pathways to the CNS (through FMT, pro-/post-biotics) may overcome these limitations and provide new perspectives for less invasive anti-obesity treatment. However, these approaches need further clinical verification.

## 5 Conclusion

We demonstrated that RYGB surgery restores leptin's anorexigenic action via amelioration of hypothalamic inflammation and ER stress in diet-induced obesity. Mechanistically, we found that RYGB interferes with hypothalamic TLR4 signaling, which most likely results from modulation of a circulating factor derived from the altered gut microbial environment upon RYGB surgery. These data advance our understanding of how gut surgery achieves sustained feeding suppression and may provide a rationale for less invasive gut-directed anti-obesity strategies.

## Authors’ contributions

J.C. designed and led the study, conducted the experiments, and wrote the manuscript. N.H., R.S., and F.S. developed and performed the surgeries. J.M., E.J., and K.S. were involved in the ex vivo analyses. S.-B.H. and M.v.B. were responsible for the microbiome profiling and data analysis. M.K.H. contributed to the study design and experiments. U.K. assisted with the study design and provided scientific input. W.K.F. conceived and directed the project, designed the experiments, supervised the participants, and wrote the manuscript.
