# Supplementary material for: Roux-en-Y gastric bypass contributes to weight loss-independent improvement in hypothalamic inflammation and leptin sensitivity through gut-microglia-neuron-crosstalk
Source: Mol Metab. 2021 Mar 16;48:101214. doi: 10.1016/j.molmet.2021.101214 (PMC8095174; doi:10.1016/j.molmet.2021.101214)
Supplement: Supplementary file 1 — Multimedia component 1 [file mmc1.docx]

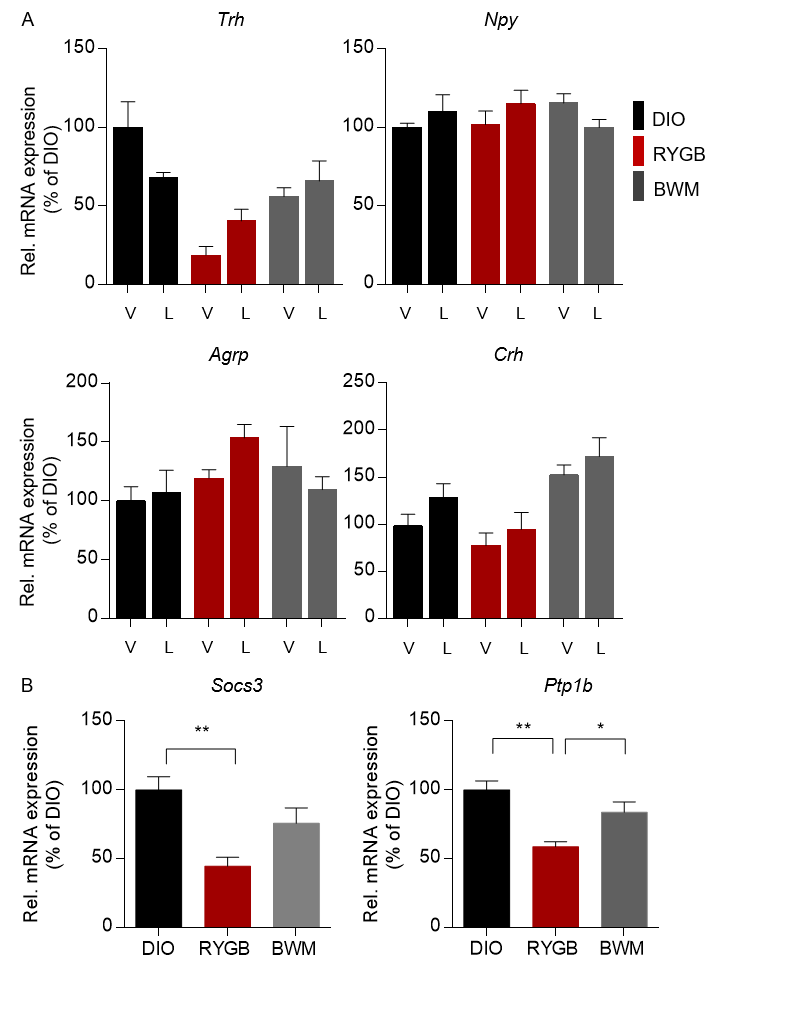


**Supplementary Figure 1. Extended data to Figure 1. RYGB surgery enhances hypothalamic leptin signaling.** (A) Hypothalamic mRNA expression of *Trh, Npy, Agrp* and *Crh* in response to leptin versus vehicle administration of RYGB- and sham-operated DIO and BWM rats at 12 weeks postopertatively. (B) Hypothalamic mRNA expression of *Ptp1b* and *Socs3*. Data are presented as mean ± SEM, (n= 4-6), with individual datapoints, **P* < 0.05, ***P* < 0.01 for the effect of any indicated comparison.


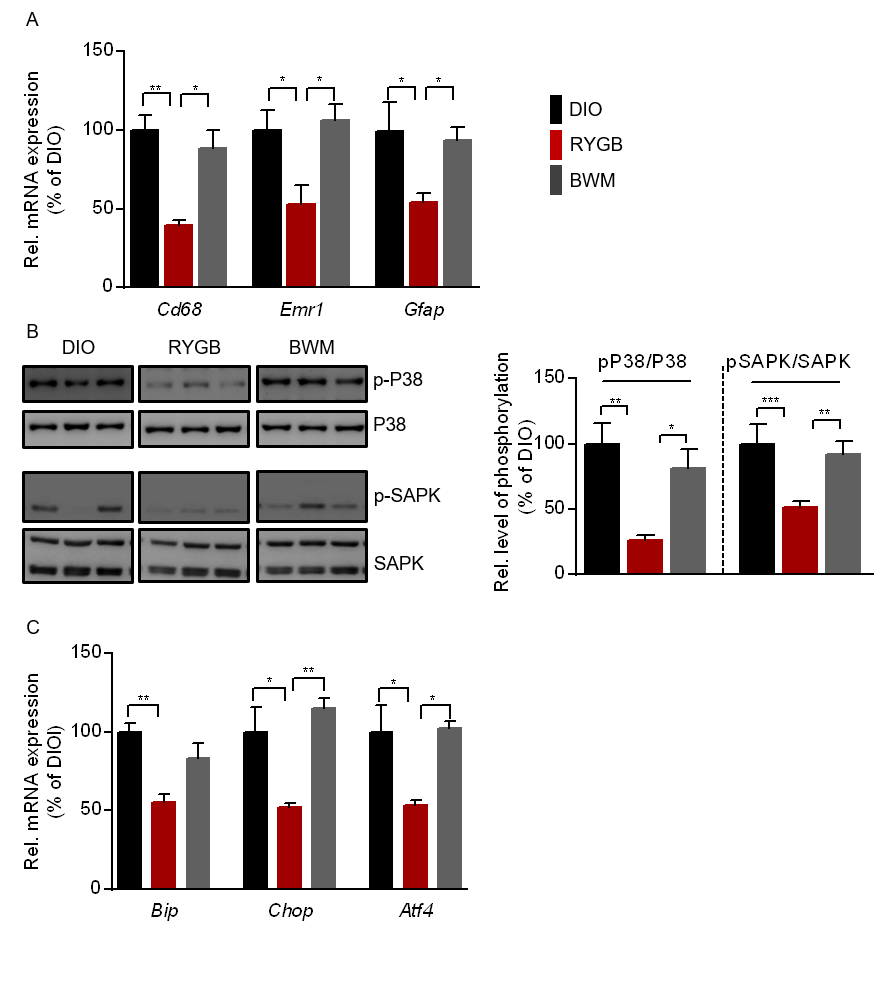


**Supplemental Figure 2. Extended data to Figure 2. RYGB surgery relieves hypothalamic gliosis, inflammatory-like response and ER stress in DIO rats.** (A) Hypothalamic mRNA expression of glial markers *Cd68*, *Emr1* and *Gfap* of RYGB- and sham-operated DIO and BWM rats at 12 weeks postoperatively. (B) Phosphorylation of p38 mitogen activated protein kinase (MAPK) and Stress-activated protein kinase (SAPK) in the hypothalamus. (C) Hypothalamic mRNA expression of ER stress markers *Bip*, *Chop* and *Atf4*. Data are presented as mean ± SEM, (n= 4-6), with individual datapoints, **P* < 0.05, ***P* < 0.01, ****P* <0.001 for the effect of any indicated comparison.

**
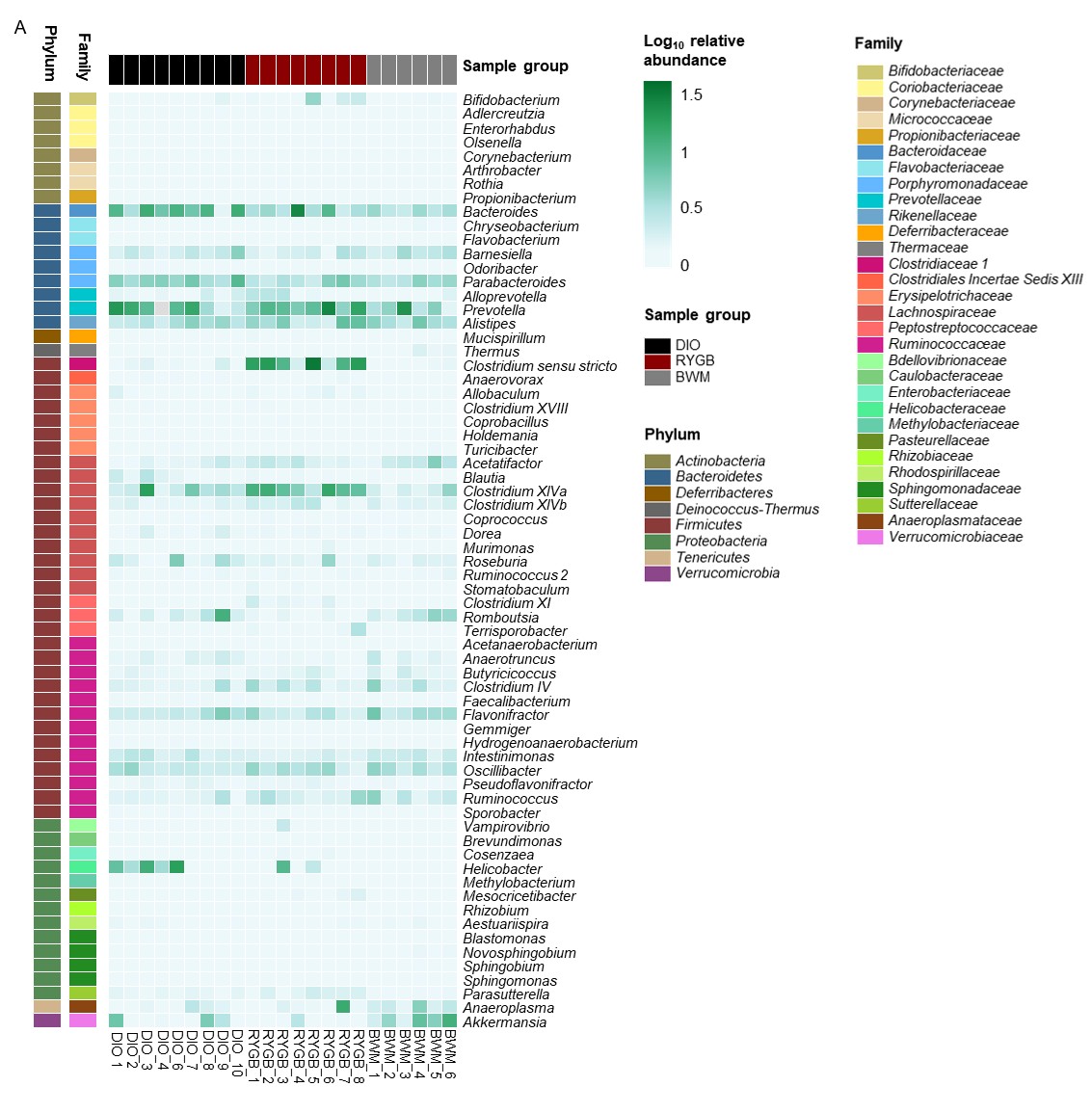
**

**Supplementary Figure 3. Extended data to Figure 5. Distinct shifts in gut microbiota composition in DIO resulting from RYGB- versus CR-induced adiposity reduction.** (A) Heatmap of relative abundance of bacterial genera from the microbiome based on read count from 16S rRNA gene sequencing from cecal content collected from animals at 12 weeks postoperatively which could be resolved to genus level. Annotation colours on the vertical axis represent the phylum (left) and the family (right) to which the genus is assigned to. Annotation colours on the top horizontal axis depict the sample group.

**Supplemental Table 1. Rat primer pairs for SYBR Green qPCRs (Part 1)**

| **Gene** | **Primer** | **Sequence (5‘-3‘)** |
| --- | --- | --- |
| *Gapdh* | Forward | AACGACCCCTTCATTGAC |
|  | Reverse | TCCACGACATACTCAGCAC |
| *Actb* | Forward | GGAGATTACTGCCCTGGCTCCTA |
|  | Reverse | GACTCATCGTACTCCTGCTTGCTG |
| *Cd68* | Forward | CTTCCCACAAGCAGCACAG |
|  | Reverse | AATGATGAGAGGCAGCAAGAGA |
| *Adgre1* (*Emr1*) | Forward | AATCGCTGCTGGCTGAATACGG |
|  | Reverse | CCAGGCAAGGAGGGCAGAGTT |
| *Gfap* | Forward | AACGACTATCGCCGCCAACTG |
|  | Reverse | CTCTTCCTGTTCGCGCATTTG |
| *Tlr4* | Forward | TCCGCTGGTTGCAGAAAATG |
|  | Reverse | GAAGTACCTCTATGCAGGGATTCAA |
| *Myd88* | Forward | TGAACTGAAGGACCGCATCG |
|  | Reverse | GCGACGACACCTTTTCTCAAT |
| *Il6* | Forward | GTGGCTAAGGACCAAGACCA |
|  | Reverse | TAGCACACTAGGTTTGCCGAG |
| *Pomc* | Forward | GCAGGGGTCTTCTCACTCCA |
|  | Reverse | GTAACTCTAAGAGGCTGGAGGTC |
| *Cartpt* | Forward | TGAGAAGGAGCTGCCAAGG |
|  | Reverse | CGG AATGCGTTTACTCTTGAGC |
| *Npy* | Forward | TAACAAACGAATGGGGCTGT |
|  | Reverse | TGTCTCAGGGCTGGATCTCT |
| *Agrp* | Forward | TGCTAGATCCACAGAACCGC |
|  | Reverse | GCAAGGTACCTGTTGTCCCA |
| *Bdnf* | Forward | TTTTCCCTCCCGGAGAGTTC |
|  | Reverse | TCCCCTTCCCTTGAGAAAGC |

**Supplemental Table 1. Rat primer pairs for SYBR Green qPCRs (Part 2)**

| **Gene** | **Primer** | **Sequence (5‘-3‘)** |
| --- | --- | --- |
| *Trh* | Forward | GCA AACTCTACCCAGCCAGT |
|  | Reverse | ATCCTAGAGTCTGCGAAGTG |
| *Crh* | Forward | GAAACTCAGAGCCCAAGTACG |
|  | Reverse | GTTTAGGGGCGCTCTCTTCT |
| *Ptpn1*  (*Ptp1b*) | Forward | GTCAGTGCAGGATCAGTGGA |
|  | Reverse | CTCCAATGTGCGTTTGGGTG |
| *Socs3* | Forward | ACCAAGAACCTACGCATCCAGT |
|  | Reverse | CTCTGACCCTTTCTTTGCTCTTTA |
| *Bip* | Forward | CTGACTTGGACACTTGGCCT |
|  | Reverse | TGAATACACCGACGCAGGAA |
| *Chop* | Forward | GCAGCGACAGAGCCAAAATA |
|  | Reverse | TTGGACCGGTTTCTGCTTTC |
| *Atf4* | Forward | CATGGCGCTCTTCACGAAAC |
|  | Reverse | GAA AAGGCATCCTCCTTGCC |

**Supplemental Table 2. Mouse primer pairs for SYBR Green qPCRs**

| *Gapdh* | Forward | GGCTGTATTCCCCTCCATCG |
| --- | --- | --- |
|  | Reverse | CCAGTTGGTAACAATGCCATG |
| *Aif1*  (*Iba1*) | Forward | GTCCTTGAAGCGAATGCTGG |
|  | Reverse | CATTCTCAAGATGGCAGATC |
| *Md2* | Forward | CGCTGCTTTCTCCCATATTGA |
|  | Reverse | CCTCAGTCTTATGCAGGGTTCA |
| *Myd88* | Forward | GTCCGACCGTGACGTCCTGC |
|  | Reverse | CCACCATGCGGCGACACCTT |
| *Cd14* | Forward | GCCAAATTGGTCGAACAAGC |
|  | Reverse | CCATGGTCGGTAGATTCTGAAAGT |
| *iNOS* | Forward | AATCTTGGAGCGAGTTGTGG |
|  | Reverse | CAGGAAGTAGGTGAGGGCTTG |
| *Nlrp3* | Forward | TGCTCTTCACTGCTATCAAGCCCT |
|  | Reverse | ACAAGCCTTTGCTCCAGACCCTAT |
| *Il18* | Forward | TGGTTCCATGCTTTCTGGACTCCT |
|  | Reverse | TTCCTGGGCCAAGAGGAAGTGATT |
| *Pomc* | Forward | TGGGCGAGCTGATGACCT |
|  | Reverse | GCCGACTGTGAAATCTGAAAGG |
| *Obrb* | Forward | ACGTGGTGAAGCATCGTACT |
|  | Reverse | GGCCATGAGAAGGTAAGGTT |
| *Bdnf* | Forward | TGCAGGGGCATAGACAAAAGG |
|  | Reverse | CTTATGAATCGCCAGCCAATTCTC |

**Supplemental Table 3. Primary antibodies used for western blotting**

| **Target** | **Host/Type** | **Company** | **Cat. No.** | **Dilution** |
| --- | --- | --- | --- | --- |
| GAPDH | Mouse mAb | Abcam | Ab8245 | 1:1000 |
| β-Actin | Rabbit pAb | Merck | A2066 | 1:500 |
| GFAP | Rabbit mAb | Abcam | ab33922 | 1:2000 |
| IBA1 | Rabbit mAb | Abcam | ab178847 | 1:1000 |
| NF-κB | Rabbit pAb | Abcam | ab16502 | 1:1000 |
| p-NF-κB | Rabbit pAb | Abcam | ab86299 | 1:1000 |
| IκBα | Mouse mAb | CST | 4814T | 1:1000 |
| p-IκBα | Rabbit mAb | CST | 2859 | 1:1000 |
| eLF2α | Rabbit mAb | CST | 5324 | 1:1000 |
| p-eLF2α | Rabbit mAb | CST | 3398 | 1:1000 |
| IRE1 | Rabbit pAb | Abcam | ab37073 | 1:1000 |
| p-IRE1 | Rabbit pAb | Novus | NB100-2323 | 1:1000 |
| STAT3 | Rabbit mAb | Abcam | ab68153 | 1:1000 |
| p-STAT3 | Rabbit mAb | Abcam | ab76315 | 1:1000 |
| ERK1/2 (P42/44) | Rabbit mAb | CST | 4295 | 1:1000 |
| p-ERK1/2  (p-P42/44) | Rabbit mAb | CST | 4370 | 1:1000 |
| SOCS3 | Rabbit pAb | CST | 2923 | 1:500 |
| PTP1B | Rabbit pAb | Abcam | ab189179 | 1:500 |
| TLR4 | Mouse mAb | Santa Cruz | sc-2930726 | 1:500 |
| Myd88 | Mouse mAb | Novus | NB100-56698 | 1:500 |
| P38 MAPK | Rabbit mAb | CST | 8690 | 1:1000 |
| p-P38 MAPK | Rabbit mAb | CST | 4511 | 1:1000 |
| SAPK | Rabbit pAb | CST | 9252 | 1:1000 |
| p-SAPK | Rabbit pAb | CST | 9251 | 1:1000 |
